# Supplementary material for: Inter-colony and inter-annual behavioural plasticity in the foraging strategies of a fjord-dwelling penguin—good news in the face of environmental change?
Source: PeerJ. 2025 Jul 7;13:e19650. doi: 10.7717/peerj.19650 (PMC12244129; doi:10.7717/peerj.19650)
Supplement: Supplemental Information 1 — Averages per colony per year are also displayed. A weight measurement was missed for one of the birds sampled from Harrison Cove in 2019. [file peerj-13-19650-s001.docx]

|  | 2019 | | 2020 | |
| --- | --- | --- | --- | --- |
|  | **Moraine** | **Harrison Cove** | **Moraine** | **Harrison Cove** |
| **Body Mass (g)** | 2500  3000  2600  2625  2850  2550 | 2975  2475  2550  2650 | 2625  2900  2700  2350  2775 | 2650  3150  3200  2325  2775  2925  2550  2775 |
| **Average (g)** | 2687.5 | 2662.5 | 2670 | 2793.75 |
