# Supplementary material for: Inter-colony and inter-annual behavioural plasticity in the foraging strategies of a fjord-dwelling penguin—good news in the face of environmental change?
Source: PeerJ. 2025 Jul 7;13:e19650. doi: 10.7717/peerj.19650 (PMC12244129; doi:10.7717/peerj.19650)
Supplement: Supplemental Information 2 — Seafloor bathymetry was downloaded from https://www.niwa.co.nz/our-science/oceans/bathymetry, salinity, and chlorophyll-a concentration from https://coastwatch.pfeg.noaa.gov, and sea surface temperature from http://marine.copernicus.eu. Salinity and temperature of fiord waters from an oceanographic mooring at Milford Marina were provided by Meridian Energy, wind speed and rainfall was taken from an electronic weather station in Milford Sound and downloaded from https://cliflo.niwa.co.nz. True spatial resolution for fiord variables could be between 0 m and 13 km since the data from a stationary mooring and weather station in Milford Marina was aligned to birds that may have been located anywhere throughout the length of the 16 km fiord. [file peerj-13-19650-s002.docx]

| Environmental variable (unit) | Spatial / temporal resolution / sensor depth (-) or height (+) | Source |
| --- | --- | --- |
| **Ocean Variables** |  |  |
| Seafloor bathymetry (m) | 250m / - / - | New Zealand Regional Bathymetry 2016 |
| Sea surface temperature (°C) | 0.01° / daily / -0.5m | Multi-scale Ultra-high Resolution (MUR) SST Analysis fv04.1 |
| Sub surface salinity (PSU) | 0.08° / daily /- 0.5m | Global_analysis_forecast_PHY_001_024 |
| Chlorophyll-a concentration (mg/m^3^) | 0.25° / daily / -0.5m | Global_analysis_forecast_BIO_001_028 |
| **Fiord Variables** |  |  |
| Salinity (PSU) | - / daily/ -0.5m | Meridian energy dataset |
| Temperature (°C) | - / daily / -0.5m | Meridian energy dataset |
| Wind speed (km/h) | - / daily / 3m | NIWA weather station |
| Rainfall (mm) | - / daily / 3m | NIWA weather station |
