# Supplementary material for: Inter-colony and inter-annual behavioural plasticity in the foraging strategies of a fjord-dwelling penguin—good news in the face of environmental change?
Source: PeerJ. 2025 Jul 7;13:e19650. doi: 10.7717/peerj.19650 (PMC12244129; doi:10.7717/peerj.19650)
Supplement: Supplemental Information 3 — Comparison of the percentage of dives made in neritic (0–200 m), shelf-slope (200–1,000 m) and oceanic waters (>1,000 m). [file peerj-13-19650-s003.docx]

|  | **2019** | **2020** |
| --- | --- | --- |
| Neritic (% dives) | 37 | 56 |
| Shelf-slope (% dives) | 21 | 20 |
| Oceanic (% dives) | 42 | 24 |
|  |  |  |
