# Supplementary material for: Inter-colony and inter-annual behavioural plasticity in the foraging strategies of a fjord-dwelling penguin—good news in the face of environmental change?
Source: PeerJ. 2025 Jul 7;13:e19650. doi: 10.7717/peerj.19650 (PMC12244129; doi:10.7717/peerj.19650)
Supplement: Supplemental Information 4 — Means were calculated using the average of individual means. Dive activity measured dives per hour during the daily foraging period between 0600 to 2000. In each Generalised Linear Mixed Model (GLMM), the random effects of Trip ID nested within Bird ID were included and an appropriate error structure was specified. p values in bold are under the set significance level of <0.05, df = 1. [file peerj-13-19650-s004.docx]

|  | 2019 | | 2020 | | Model Outputs | | | | | | | |
| --- | --- | --- | --- | --- | --- | --- | --- | --- | --- | --- | --- | --- |
| Response Variable | Moraine | Harrison Cove | Moraine | Harrison Cove | Explanatory Variables | Model | Estimate | CI (lwr, upr) | | S.E. | *X*^2^ value | *p* value |
| Dive activity (dives h^-1^) | 45.9 ± 11.3 | 38.9 ± 8.8 | 33.7 ± 7.6 | 39.4 ± 8.8 | Site (MO)  Year (2020)  Site (MO):Year (2020) | GLMM – Negative Binomial | 0.040  0.045  -0.043 | -0.040  -0.034  -0.123 | 0.119  0.125  0.036 | 0.041  0.041  0.041 | 0.952  1.25  1.13 | 0.329  0.263  0.288 |
| Number of wiggles per dive | 7.6 ± 2.2 | 7.2 ± 2.8 | 9.2 ± 1.8 | 8.9 ± 1.4 | Site (MO)  Year (2020)  Site (MO):Year (2020) | GLMM -Negative Binomial | -0.015  -0.107  -0.011 | -0.090 -0.183  -0.086 | 0.061  -0.031  0.065 | 0.039  0.039  0.039 | 0.142  7.66  0.080 | 0.706  **0.006**  0.777 |
| Dive duration (s) | 64.4 ± 17.0 | 71.1 ± 20.5 | 89.0 ± 19.3 | 66.7 ± 12.4 | Site (MO)  Year (2020)  Site (MO):Year (2020) | GLMM - Gaussian | -0.047  -0.066  0.088 | -0.136  -0.155  -0.000 | 0.041  0.022  0.177 | 0.045  0.045  0.045 | 1.09  2.15  3.81 | 0.297  0.143  0.051 |
| Max dive depth (m) | 19.0 ± 5.7 | 23.0 ± 7.8 | 30.9 ± 8.7 | 16.1 ± 5.2 | Site (MO)  Year (2020)  Site (MO):Year (2020) | GLMM - Gamma | -0.113  -0.032  0.197 | -0.230  -0.149  0.080 | 0.004  0.085  0.314 | 0.060  0.060  0.060 | 3.57  0.284  10.91 | 0.059  0.594  **<0.001** |
| Descent rate (ms^-1^) | 0.86 ± 0.11 | 0.90 ± 0.16 | 1.05 ± 0.08 | 0.85 ± 0.07 | Site (MO)  Year (2020)  Site (MO):Year (2020) | GLMM - Gaussian | -0.037  -0.038  0.066 | -0.068  -0.069  0.034 | 0.007  0.006  0.109 | 0.019  0.019  0.019 | 3.77  4.07  12.09 | 0.052  **0.044**  **<0.001** |
| Ascent rate (ms^-1^) | 0.84 ± 0.03 | 0.86 ± 0.06 | 0.91 ± 0.08 | 0.83 ± 0.08 | Site (MO)  Year (2020)  Site (MO):Year (2020) | GLMM - Gaussian | -0.008  -0.015  0.022 | -0.036  -0.043  -0.006 | 0.020  0.013  0.050 | 0.014  0.014  0.014 | 0.286  1.08  2.33 | 0.593  0.298  0.127 |
| Bottom time (s) | 30.5 ± 8.7 | 32.3 ± 9.5 | 40.5 ± 8.9 | 35.2 ± 4.3 | Site (MO)  Year (2020)  Site (MO):Year (2020) | GLMM - Gaussian | -0.020  -0.092  0.041 | -0.104  -0.176  -0.043 | 0.064  -0.008  0.126 | 0.043  0.043  0.043 | 0.212  4.64  0.935 | 0.645  **0.031**  0.334 |
| Foraging efficiency | 0.37 ± 0.03 | 0.38 ± 0.02 | 0.40 ± 0.03 | 0.43 ± 0.03 | Site (MO)  Year (2020)  Site (MO):Year (2020) | GLMM - Gaussian | 0.032  -0.041  -0.020 | -0.003  -0.075  -0.055 | 0.067  -0.006  0.014 | 0.018  0.018  0.018 | 3.36  5.31  1.33 | 0.067  **0.021**  0.249 |
