# Supplementary figures and images for: Inter-colony and inter-annual behavioural plasticity in the foraging strategies of a fjord-dwelling penguin—good news in the face of environmental change?
Source: PeerJ. 2025 Jul 7;13:e19650. doi: 10.7717/peerj.19650 (PMC12244129; doi:10.7717/peerj.19650)

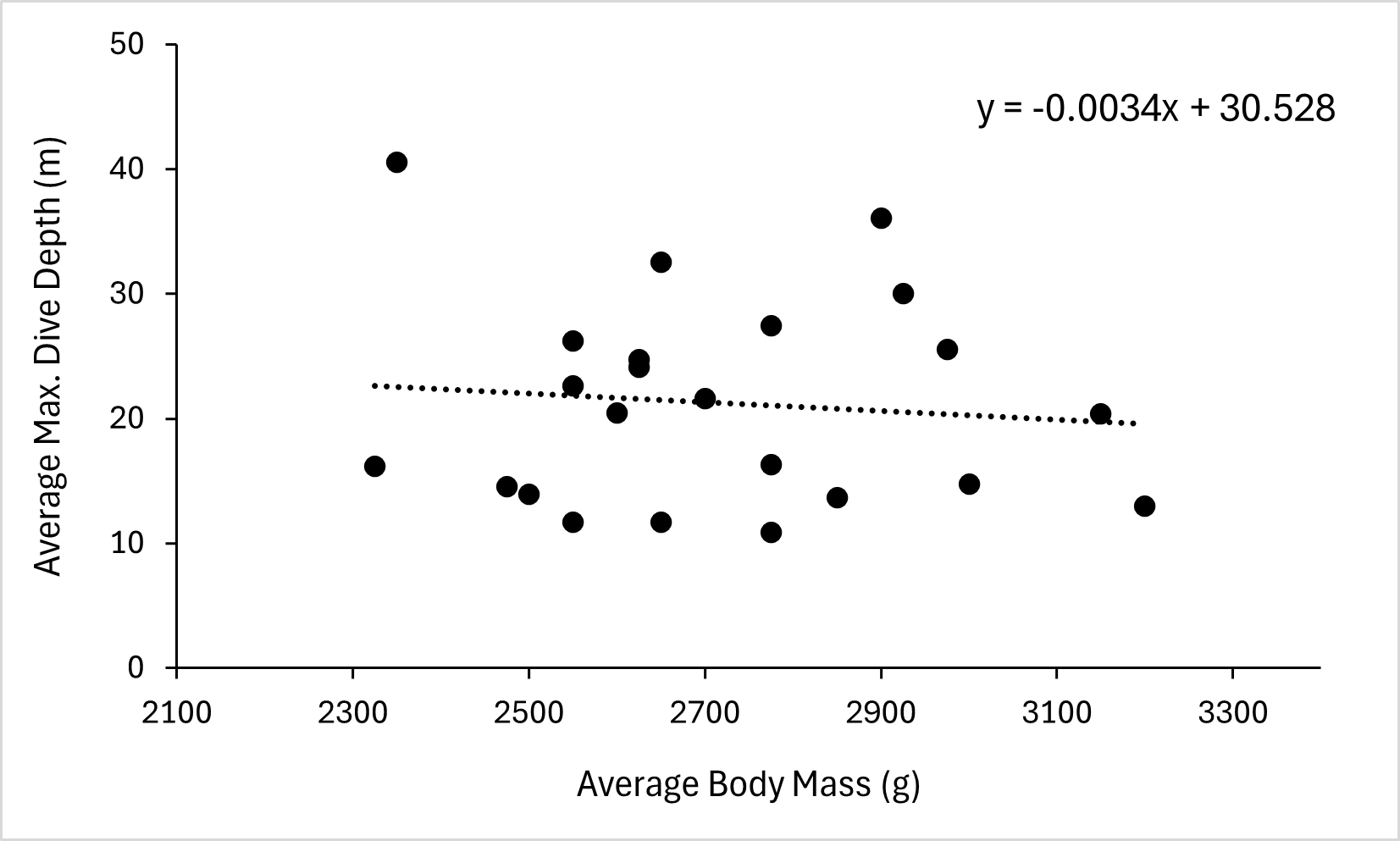

Supplement: Supplemental Information 5 [file peerj-13-19650-s005.png]

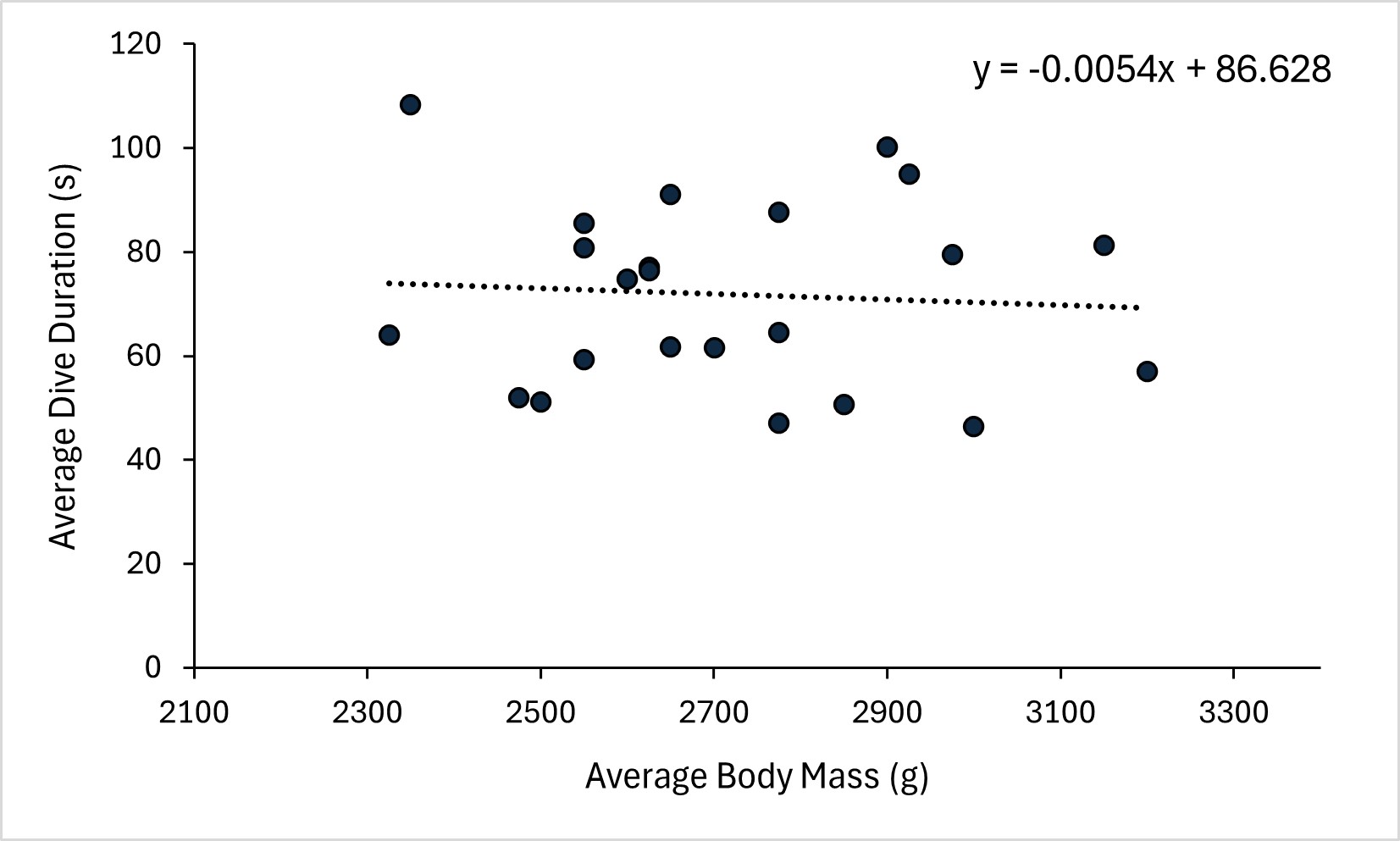

Supplement: Supplemental Information 6 [file peerj-13-19650-s006.jpg]
